# Regional Societies: Fostering Competitive Research Through Virtual Infrastructures

**DOI:** 10.1371/journal.pbio.0020372

**Published:** 2004-12-14

**Authors:** Steven F Jennings, Andrey A Ptitsyn, Dawn Wilkins, Russel E Bruhn, William Slikker, Jonathan D Wren

## Abstract

The MidSouth Computational Biology and Bioinformatics Society (MCBIOS) describes its efforts to provide local opportunities for researchers to learn and connect with colleagues

The rich get richer while the poor get poorer. This is as true for research in the life sciences as it is for society in general. Why is this? To put it simply: because existing resources can be leveraged to generate new ones. In life science research, these resources are often expressed in terms of people, equipment, and funding. Those who have more in these areas—large research organizations, support staff, extensive facilities, and pipelines of funding—can be more productive, and thereby more successful in maintaining and even expanding their infrastructure. In the United States, this “scientific wealth” is concentrated in certain geographic areas, particularly along the east and west coasts. While there are many highly competent individuals in other, less wealthy, regions, they often struggle to attract funding because they lack this infrastructure.

Fortunately, scientific progress is also made through collaboration and cooperation—activities fostered through the exchange of viewpoints and the exploration of ideas. Given a framework in which this networking can occur, some of the problems that result from a lack of infrastructure can be overcome. By definition, collaboration involves the sharing of ideas, capabilities, and resources. While it may not be possible to access all necessary resources locally, finding partners—who have their own local capabilities and resources—can enhance one's own research environment. Through these kinds of efforts, “virtual infrastructures” grow. And through collaborative efforts, those of us in some of the “poorer” regions of the United States have opportunities to successfully compete for national sources of funding by becoming better able to address the “existing research environment” consideration on most grant applications.[Fig pbio-0020372-g001]

**Figure pbio-0020372-g001:**
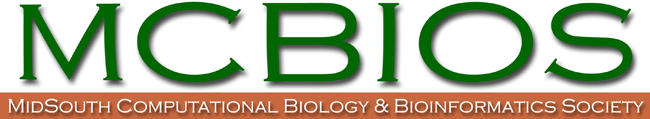


Building a virtual infrastructure is the primary purpose behind the formation, in the United States, of the MidSouth Computational Biology and Bioinformatics Society (MCBIOS; www.MCBIOS.org), created to serve a geographical area that includes Arkansas, Louisiana, western Tennessee, Missouri, Mississippi, Oklahoma, and east Texas. By its very nature, bioinformatics involves people from many different backgrounds and is therefore ideal for collaborative efforts. Like many other regional societies, our primary goal is to provide a framework in which collaboration and cooperation can occur. By sponsoring regional activities (at the present time, primarily our annual conference) we hope to bring educators, researchers, and especially students together with others who have similar and/or complementary interests. Through the society, not only can medical scientists make contact with computational experts, but researchers in Arkansas can connect with researchers in Oklahoma, educators from Missouri can interact with educators from Louisiana, and students from Mississippi can find others from Tennessee who are working in the same area. Communication across specialties and areas of expertise is especially important for fostering interdisciplinary efforts and exposing students to a broader range of topics than might be available at their own institution. Nowhere is this truer than in the application of informatics to a variety of disciplines.[Fig pbio-0020372-g001]

Regional societies need not be in competition with national or international societies. In fact, many of these larger organizations are finding that it is to their advantage to encourage close relationships with their regional counterparts. The International Society of Computational Biology (ISCB; www.ISCB.org), for example, has recognized MCBIOS under their “regional affiliate” program. Likewise, MCBIOS is encouraging its members to form “local chapters” (for example, the Oklahoma chapter of MCBIOS, www.OKBIOS.org). These local chapters are eligible to host the annual MCBIOS conference and are able to support even closer interactions among their local participants. The hierarchy of affiliations provides an abundance of opportunities for members to participate based upon their interests, financial resources, and tolerance for travel.

Regional events are by nature more inclusive, since smaller investments of time and money are required to participate. This, in turn, creates a more diverse group of attendees, since it enables those whose primary research or educational focus may be outside the subject matter to participate. While not everyone in our region may be able to attend the annual ISCB conference (in 2004 it was held in Glasgow, Scotland), we have deliberately chosen locations for the MCBIOS annual conference that would be within a day's drive for all members. At the inaugural 2003 MCBIOS conference, for example, with a theme of “Building Networks,” a number of computer scientists attended who were able to find out more about bioinformatics and their possible contribution to life science research; they would probably not have been able to make the investment to attend a national or international meeting on bioinformatics.

While still maintaining high standards, regional events also increase the number of venues for the presentation of research results and creative efforts in the educational arena. While the ISCB program, which is already highly self-selecting, accepts less than 15% of their submissions, almost all submissions at the first annual MCBIOS conference could be accommodated, at least in poster form. These regional events can also attract well-respected keynote speakers and provide training opportunities that might not otherwise be available to the membership. For example, Dr. Alan Leshner, CEO of the American Association for the Advancement of Science, gave the keynote address of the 2004 MCBIOS conference. In 2003, we were also able to host a special training session by the National Center for Biotechnology Information on their GenBank and molecular biology tools.

While MCBIOS is still new, we have plans to extend our activities—and thus our impact—beyond our annual conference. We are dedicated to supporting our local chapters, and have plans to develop a speakers' bureau to serve them. Plans to collaborate on regional technology efforts (e.g., a regional computing grid) and on multi-institutional educational programs are also in the works. Through the auspices of the society, we hope to increase regional credibility and attract national funding in support of these infrastructure improvements.

In the long term—and in addition to supporting the intellectual efforts of our members through a vibrant organizational community—our goal is to increase extramural funding for our represented institutions. We hope to achieve this by fostering competitive research. And this competitive research will be possible on a larger scale through our development of a virtual infrastructure—one that comes about through regional collaboration and a pooling of resources. In conjunction with other infrastructure-building efforts (such as the Biomedical Research Infrastructure Network program sponsored by the National Institutes of Health's National Center for Research Resources), we hope to see externally funded research in the life sciences significantly increase within our region, the American “Midsouth.”

(The 2003 and 2004 MCBIOS conferences have been supported in part by National Institutes of Health grant P20 RR-16460 from the Arkansas Biomedical Research Infrastructure Network Program [http://BRIN.UAMS.edu] of the National Center for Research Resources through a grant to underwrite student participation and awards at the conferences. The 2004 MCBIOS conference was also supported in part by a grant from the National Center for Toxicological Research [www.FDA.gov/NCTR].)

## Abbreviations

ISCB: International Society of Computational Biology
MCBIOS: MidSouth Computational Biology and Bioinformatics Society

